# Bidirectional-Reinforced Carbon Fiber/Polyether-Ether-Ketone Composite Thin-Walled Pipes via Pultrusion-Winding for On-Orbit Additive Manufacturing

**DOI:** 10.3390/ma17020293

**Published:** 2024-01-06

**Authors:** Yuanhao Xia, Long Jiang, Yi Chen, Yiping Zhao, Lili Yang, Dengteng Ge

**Affiliations:** 1 State Key Laboratory for Modification of Chemical Fibers and Polymer Materials, College of Materials Science and Engineering, Donghua University, Shanghai 201620, China; 2200353@mail.dhu.edu.cn; 2 Institute for Engineering and Technology (Shanghai), Xinxing Cathay International Group, Shanghai 201403, China; jianglong840@163.com (L.J.); yping2017@163.com (Y.Z.); 3 Beijing Spacecrafts, China Academy of Space Technology, Beijing 100094, China; chenyicast529@163.com

**Keywords:** on-orbit additive manufacturing, CF/PEEK, biaxial reinforcement, thin-walled pipe

## Abstract

Benefitting from lightweight, high strength, long life, and green recyclability, continuous fiber reinforced thermoplastic composite (CFTPC) pipes have attracted extensive interest, especially in the on-orbit additive manufacturing of structural components. However, the preparation of CFTPC pipes remains challenging due to the on-orbit limited space and high processing temperature of thermoplastic resin. Here, we report an effective approach for high performance carbon fiber/polyether-ether-ketone (CF/PEEK) thin-walled pipes via bidirectional reinforcement using the pultrusion-winding technique. The continuous fabrication of thin-walled pipes can be achieved, but the limitation by the size of core mold is also broken. The compressive and shear performance of CF/PEEK pipes with different layer designs have been studied based on experiments and simulations. With the increase in axial prepreg tape layer, the resultant CF/PEEK pipes exhibit greatly improved axial compression strength. The finite element analysis indicates that the maximum axial stress is decreased due to the axial enhancement. The flexural strength is greatly proved with pultrusion–winding cycles. The simulation confirms that the circumferential strain is effectively reduced. The high performance of bidirectional reinforced CF/PEEK pipes and the facile controllability of this approach highlight their suitability for utilization in on-orbit manufacturing of large-scale structures.

## 1. Introduction

Utilizing portable materials or extraterrestrial resources in space, on-orbit additive manufacturing technology has attracted extensive interest due to the in situ manufacturing of target products outside of Earth. With the increasing demand for large-scale structures such as space solar power plants, on-orbit additive manufacturing technology is considered one of key strategic technologies to enhance human space-activity capabilities, ensuring the construction of extraterrestrial bases and deep space exploration [1,2]. Between 2014 and 2016, NASA and the Made in Space (MIS) sent second-generation additive manufacturing equipment to the International Space Station twice to explore the additive manufacturing process under microgravity conditions [3,4]. Due to the advantages of lightweight, high strength, and flexible assembly, the truss structure composed of continuous fiber-reinforced composite pipes/rods is essential to numerous practical applications shown in space stations, space telescopes, large antennas, general aircraft, and satellites [5,6,7,8]. In 2013, Tethers Unlimited proposed the concept project “SpiderFab”, which combines 3D printing technology with the aerospace industry to manufacture large space structures in space like a spider web [9]. Compared with thermosetting composites, the thermoplastic composites have shown higher strength, longer life, better corrosion resistance and green recyclability [10,11,12,13,14,15,16]. The applications of high-performance thermoplastic resins such as PEEK have been widely studied. Zhao et al. [17] prepared low melting point carbon fiber-reinforced composite laminates using PAEK, then injected high melting temperature PEEK and short fiber reinforced PEEK resin. The influence of molding temperature on interface strength was explored. Choi et al. [18] prepared CF reinforced PEEK resin composites under different automatic fiber placement laser powers and studied their bonding and mechanical properties after induction welding.

However, the on-orbit preparation of continuous fiber-reinforced thermoplastic composite pipes/rods is still currently challenging as a result of two reasons. Firstly, thermoplastic resins exhibit high processing temperature, high viscosity, and poor flowability. Secondly, the space station shows complex environments of limited space and microgravity in space. At present, there are two categories for the fabrication of continuous fiber-reinforced composite pipes: winding and extrusion forming [19,20]. Laser-assisted winding (LATW) is a highly automated process that can be used to manufacture tubular fiber-reinforced thermoplastic composites. For example, Hosseini et al. [21] studied the critical evaluation of temperature evolution on the substrate and tape surfaces during the LATW process of CF/PEEK pipes and optimized the preparation process. However, the length of the pipes prepared by this process was limited by the size of core mold. Moreover, this technique is not suitable for continuous production, and only circumferential reinforcement is available, leading to poor axial performance. Chen et al. [22] developed a new type of thermoplastic-reaction injection extrusion test line and studied and optimized process parameters such as injection zone temperature, extrusion mold temperature, and extrusion speed. They successfully prepared fiber-reinforced thermoplastic composites continuously. However, only the axial strength was enhanced in this process. There is still room for improvement in mechanical properties. Furthermore, 3D printing is an important process in space in orbit manufacturing. The assembly of 3D-printed composite materials has a wide range of applications within in-orbit manufacturing and the in situ resource utilization of planetary surfaces [23]. McNiffe et al. [24] used a specially improved and optimized 3D printer to manufacture functionally graded polyether-ether-ketone components. Santiago et al. [25] provided a comprehensive mechanical analysis and CT-scan-based dimensional study of carbon fiber PEEK lattice structures enabled with high-temperature support and including model validation. However, 3D printing is associated with problems of low processing efficiency and small processing space, making it difficult to process large-sized structures.

To address these problems, in this paper we propose a pultrusion-winding technique for thermoplastic composite pipes with bidirectional reinforcement, which achieves continuous forming of pipes. Based on CF/PEEK prepreg tapes, CF/PEEK pipes were prepared with different layer number of axial prepreg tape and pultrusion–winding cycles of circumferential tapes. Experimental and simulation studies were conducted on the mechanical properties of these pipes. We believe that our research provides experimental basis for space on-orbit additive preparation.

## 2. Materials and Methods

### 2.1. Materials 

The TU200 series carbon fiber-reinforced polyether-ether-ketone composite prepreg tapes were provided by Barrday Company (Cambridge, ON, Canada). The resin content of the prepreg tape was 34%, the glass transition temperature (Tg) was 143 °C, the density was 1.58 g/cm^3^, and the areal weight of the prepreg was 145 g/m^2^.

### 2.2. Preparation of CF/PEEK Pipes

**Figure 1:**
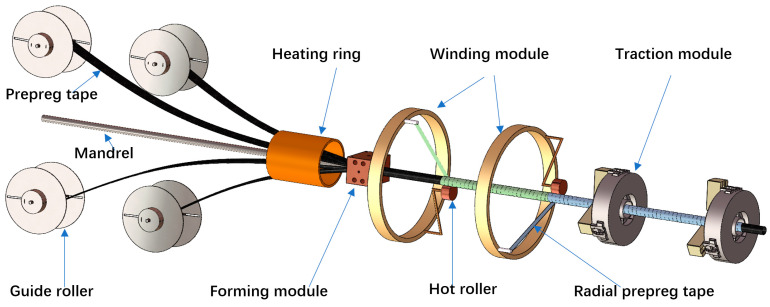
Schematic of the self-designed pultrusion-winding equipment for composite pipes.

Based on the reinforcement design concept of both axial and circumferential direction, the pultrusion-winding forming equipment was successfully developed in our lab. As shown in Figure 1, the fabrication equipment of CF/PEEK pipes mainly included three parts: the extrusion module, winding module, and traction module. In the extrusion module, the prepreg tape (width = 80 mm) was softened by heating with a heating ring, and then rolled into an axially reinforced inner lining pipe through a forming mold. In the winding module, under the action of hot roller, the narrow prepreg tape (width = 6.35 mm) was wound and welded in situ onto the inner lining pipe. The traction module continuously pulled forward to achieve continuous molding.

In this method, the core mold was fixed, and the inner axial reinforcement layers were wrapped around the core mold. The inner axial reinforcement layers were pulled outward through an auxiliary traction pipe. During the traction process, winding reinforcement was carried out, and the winding composite pipe was pulled to a position that could be clamped by the traction mechanism before removing the auxiliary traction pipe. The traction mechanism directly clamps the composite pipe fittings, achieving synchronous winding reinforcement and forward pulling demolding. During this process, the core mold remains fixed and the pipe fittings are simultaneously wound and demolded outward, achieving continuous molding. The length of this process pipe fittings was only limited by the length of the prepreg tape.

### 2.3. Design of Different Pipe Fitting Samples

In order to investigate the effects of different axial and circumferential structural designs on the mechanical properties of composite pipes, we changed the layer number of axial prepreg tape and bidirectional reinforcement layers. As shown in Table 1, different pipes with different structures are exhibited: [0_2_/84.2/−84.2], [0_3_/84.2/−84.2], [0_4_/84.2/−84.2], [0/84.2/−84.2]_2_, and [0/84.2/−84.2]_4_. The sample pipes were labeled as Sample A, Sample B, Sample C, Sample D, and Sample E, respectively.

### 2.4. Characterization

The morphologies were observed using scanning electron microscope (S4800, Hitachi, Tokyo, Japan). The thermal properties were tested using a thermogravimetric analysis (Perkin Elmer, Waltham, MA, USA) and differential scanning calorimetry (CLARUS SQ8-STA8000, Perkin Elmo, Waltham, MA, USA).

The radial compression stiffness of the pipes was tested using a universal testing machine (WDW3020, Changchun Kexin, Changchun, China). The specimen was placed horizontally between two parallel pressure plates with a loading speed of 10 mm/min. The radial compression stiffness *E_c_* can be calculated by

(1) $$E_{C}=\frac{F_{1}}{∆Y}$$

where *F*_1_ is the radial compression line load and ∆*Y* is the variation of the pipe diameter. Here, the radial compression line load represents the force acting on a pipe fitting per unit length. It is the ratio of load to the length of the pipes. The axial compression performance of pipes was also characterized at a rate of 5 mm/min. The axial compression strength σ*_c_* can be calculated by

(2) $$\sigma_{c}=\frac{4F_{2}}{\pi (D^{2}\;-\;d^{2})}$$

where *F*_2_, *D*, and *d* indicate the failure load, the average outer, and inner diameter of pipe, respectively. The interlayer shear strength of the pipes was also tested using the universal testing machine (WDW3020, Changchun Kexin, China). The center of the sample was placed perpendicular to the indenter, and the experimental speed was 2 mm/min until the sample was damaged. The interlayer shear strength *τ_s_* can be calculated by

(3) $$\tau_{s}=0.75\times \frac{P_{m}}{b\times h}$$

where *P_m_* is the failure load, *b* is the width of the sample, and *h* is the thickness of the sample.

### 2.5. Finite Element Simulation Analysis

To verify the effect of biaxial reinforcement layer method on the mechanical properties of pipes, the finite element simulation software Abaqus was used to establish pipe fitting models with different layer methods. Static analysis was conducted to verify the influence of different layer methods on the mechanical properties of pipes. The material type was selected as Lamina and the mechanical performance parameters of the CF/PEEK prepreg tape were inputted, as shown in Table 2. Shell unit was created and composite layers were set to complete the establishment of composite pipes with different layers.

## 3. Results and Discussion 

### 3.1. Optimization of Fabrication Parameters

**Figure 2:**
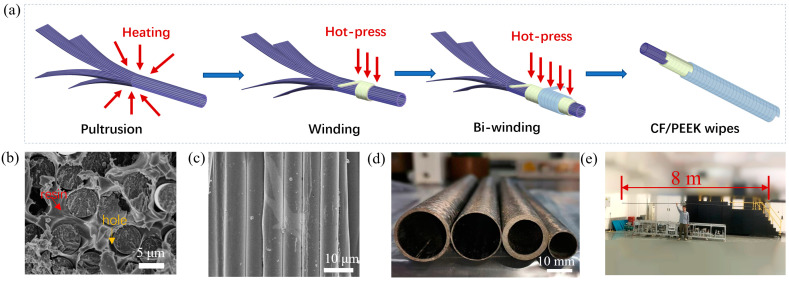
(**a**) Schematic for the forming of CF/PEEK pipes based on prepreg tapes. (**b**) Cross-sectional SEM image of prepreg tape. (**c**) Surface SEM image of prepreg tape. (**d**) Image of prepared composite pipes. (**e**) Photos of CF/PEEK pipe with a length of 8 m and the fabrication equipment.

As shown in Figure 2a, the pultrusion-winding technique mainly includes two steps: the rolling and forming of pipes under the heating and softening of prepreg tapes, and circumferential enhancement via winding under hot pressing. In the first step, wide tapes (width = 80 mm) were used to reinforce the axial performance, while narrow tapes (width = 6.35 mm) were used for winding step. From the cross-sectional SEM image of prepreg tape (Figure 2b), most PEEK resin was firmly coated on the surface of CF, but there remained some holes, indicating a moderate interfacial compatibility between CF and PEEK resin. Meanwhile, the surface SEM image of prepreg tape (Figure 2c) shows that fibers are arranged neatly and resin is uniformly wrapping the fibers without wrinkles, bubbles or white spots. The fiber arrangement was smooth, without stacking or bending, and was suitable for the continuous production of composites. By adjusting the layer number of axial prepreg tape and winding cycles of circumferential prepreg tape, pipes with different structures and wall thickness can be fabricated (Figure 2d). Figure 2e exhibits a CF/PEEK pipe with a length of 8 m, thickness of 0.6 mm, and outer diameter of 20 mm. The length/diameter was up to 400 while the high size accuracy indicated the bidirectional enhancement of composite pipes.

**Figure 3:**
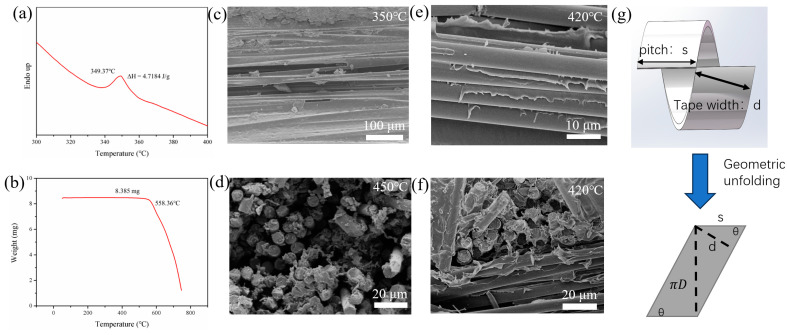
(**a**) DSC curve of CF/PEEK prepreg tape. (**b**) TGA curve of CF/PEEK prepreg tape. (**c**) Surface SEM image of composite pipe with a forming temperature of 350 °C. (**d**) The cross-sectional SEM image of pipe with forming temperature of 450 °C. (**e**) Surface image and (**f**) cross-sectional SEM images of pipe with a forming temperature of 420 °C. (**g**) Geometric structure of the prepreg tape during circumferential winding.

During the winding process, parameters such as the forming temperature and winding angle should be optimized to achieve the best interfacial bonding and composite performance. Differential scanning calorimeter (DSC) curves of the prepreg tape were firstly tested as shown in Figure 3a. The endotherm peak proves that the melting temperature of PEEK resin is 349.37 °C. Thermogravimetric analysis (TGA) was also performed on the prepreg tape, as shown in Figure 3b. When the temperature exceeded 558.36 °C, PEEK began to show significant weight loss, indicating the decomposition of PEEK. Based on the DSC and TGA results, it was preliminarily determined that the forming temperature of the CF/PEEK prepreg tape was within the range of 350–500 °C. The CF/PEEK pipes were formed under different pultrusion temperatures. Figure 3c,d shows the SEM images of the composites. When the molding temperature was low (350 °C), pores and voids appeared on the surface of pipe. Due to insufficient resin flow, gaps were left in the prepreg tapes during folding and synthesizing. When the mold temperature was high (450 °C), fibers on the cross-sections were surrounded by a large amount of PEEK resin, which reflected that the PEEK matrix was integrated with fiber tightly. Thus, the resin was fully melted at a high pultrusion temperature. However, it was found that some resin remained on the inner wall of mold, and partial carbonization was detected. The resin content decreased, and there were gaps between fibers, resulting in the appearance of pores on the surface of the prepreg tape folding-composite molding rod. The final molding temperature was determined to be 420 °C. The SEM images of the formed pipe at this temperature are shown in Figure 3e,f. No resin accumulation could be found in the mold wall. Moreover, the resin bonding between the fibers in both directions was relatively uniform. The surface of the pipe was uniformly coated with a layer of resin, indicating sufficient resin flow and improved interfacial compatibility between CF and PEEK. 

In order to determine the winding angle, the geometric structure of the winding tape is shown in Figure 3g. In the case where the tape perfectly overlaps, the winding angle *θ* was determined by the winding bandwidth and pipe diameter:

(4) $$\tan\theta =\frac{\pi D}{s}=\frac{\pi D\sin\theta }{d}$$

where *D*, *d*, and *s* indicate the diameter of the pipe, the winding bandwidth, and the pitch, respectively. If the width of the prepreg tape is 6.35 mm while the diameter of pipe is about 20 mm, the winding angle is thus approximately 84.2°.

**Figure 4:**
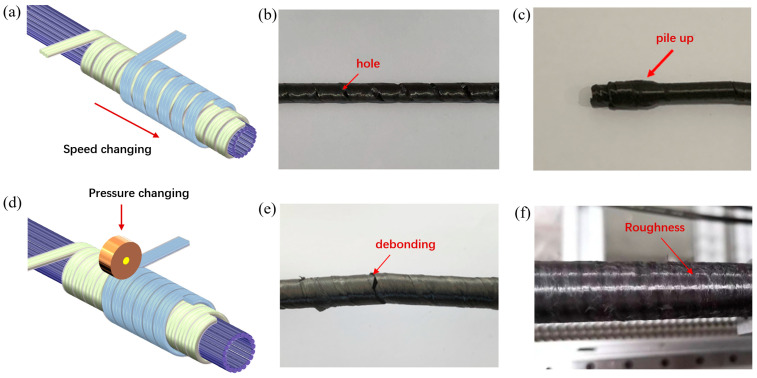
(**a**) Schematic diagram of speed control in winding process. (**b**) Holes on the surface of pipe. (**c**) The accumulation of prepreg tape. (**d**) Schematic diagram of hot roller pressure and temperature control. (**e**) Debonding occurs between layers of prepreg. (**f**) Rough surface of pipe.

Further, improper process parameters can lead to defects in the pipes. As shown in Figure 4a–c, when the traction speed is too fast, the winding reinforcement prepreg cannot be tightly connected, and holes appear on the surface of the pipes. When the traction speed is too slow, the winding reinforcement prepreg repeatedly winds in a small space, causing stacking of the prepreg tape and uniformity of the pipe. The pressure and temperature of the hot roller can also affect the quality of the pipes, as shown in Figure 4d–f. When the pressure and temperature of the hot roller are low, there is debonding between the prepreg, and the interface bonding performance is poor. However, when the pressure and temperature are high, the surface of the pipe is damaged, resulting in a rough surface. Finally, the winding speed coordination was obtained based on the winding angle. The optimal molding process was obtained with a winding speed of 4.7 rpm/min, a traction speed of 30 mm/min, a winding pressure of 0.2 MPa, and a molding temperature of 420 °C.

### 3.2. Effect of Axial Layer Number on Mechanical Properties of CF/PEEK Pipes 

**Figure 5:**
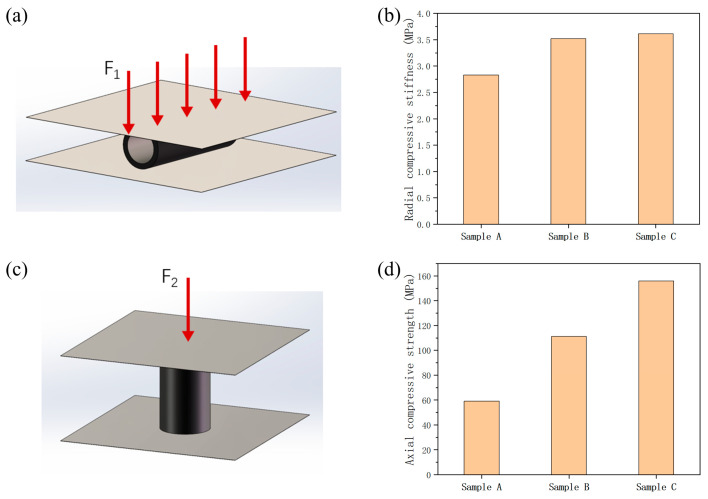
(**a**) The radial compression test method. (**b**) The radial compression test results. (**c**) The axial compression test method. (**d**) The axial compression test results.

In order to investigate the effect of axial layer number on the mechanical properties of CF/PEEK composite pipes, circumferential and axial compression tests were conducted on Samples A, B, and C. The radial compression test method is shown in Figure 5a, and the test results are shown in Figure 5b. With the increase in the axial layer number of prepreg tapes, the radial compression stiffness is slightly increased. The radial compression stiffness of Sample C was 3.61 MPa, which was 27.5% higher than that of double-layer axial prepreg tapes pipe.

The axial compression test method is shown in Figure 5c, and the test results are shown in Figure 5d. With the increase in the axial layer number of prepreg tapes, the axial compression strength of the pipes exhibits significant improvement. The axial compression strength of Sample C was 155.93 MPa, which was 163.4% higher than that of double-layer axial prepreg tapes pipe, respectively. The axially distributed carbon fiber effectively shared compression stress, resulting in a significant improvement in the axial performance of the pipes.

**Figure 6:**
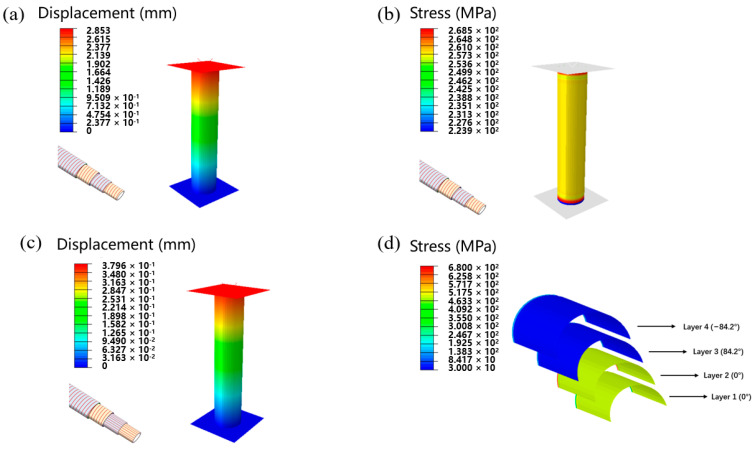
(**a**) Axial compression displacement simulation result of winding pipe. (**b**) Axial compression stress simulation result of winding pipe. (**c**) Axial compression displacement simulation result of biaxial reinforced pipe. (**d**) Axial compression stress simulation result of different layers of biaxial reinforced pipe.

To better understand the role of axial fiber, finite element simulation was applied to analyze the microstructural stress and displacement distribution. The boundary conditions were set, then the plate on the bottom of the pipe was fixed completely, and a pressure load of 10,000 N to the upper plate was applied. The traditional winding tube of [84.2/−84.2/84.2/−84.2] was also simulated to compare a bidirectional reinforced tube with the same number of layers with Sample A. As shown in Figure 6a–d, according to the simulation results, it can be seen that under the same load, Sample A has a smaller displacement compared to conventional wrapped pipe, exhibiting better axial compression performance. Through stress simulation result, it can be seen that the compression stress withstood by the axial layer was 3–4 times of that of the circumferential layer in Sample A. Therefore, the axial reinforcement layer bears a greater part of the axial pressure.

**Figure 7:**
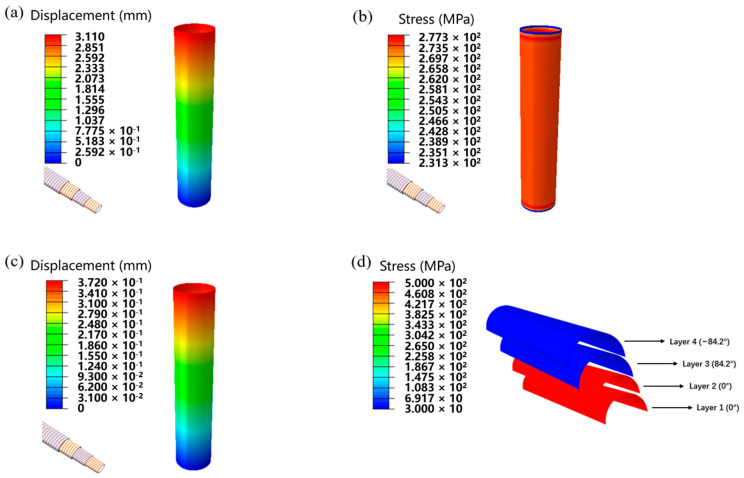
(**a**) Tensile displacement simulation result of winding pipe. (**b**) Tensile stress simulation result of winding pipe. (**c**) Tensile displacement simulation result of biaxial reinforced pipe. (**d**) Tensile stress simulation result of different layers of biaxial reinforced pipe.

The tensile performance of pipes was also simulated. We set boundary conditions, fixed the bottom of the pipe completely, and applied a tensile load of 10,000 N to the upper of the pipe. As shown in Figure 7a–d, according to the simulated results, it can also be seen that under the same tensile load, the displacement of Sample A was smaller than that of traditional wrapped pipes, exhibiting better tensile resistance. From the stress simulation result, it can be seen that the tensile stress borne by the axial layer in Sample A was much higher than that of the circumferential layer. Therefore, the axial reinforcement layer bears a significant tensile force.

### 3.3. Effect of Pultrusion–Winding Cycles on Mechanical Properties of CF/PEEK Pipes

**Figure 8:**
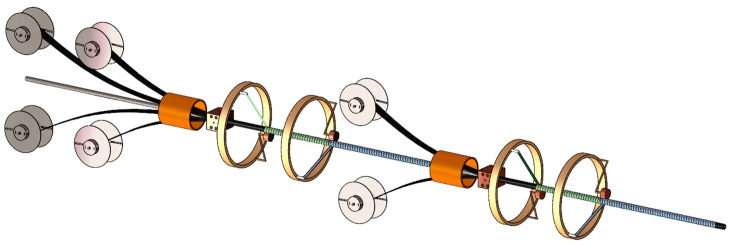
Schematic for the multi-level pultrusion-winding equipment.

Moreover, the pultrusion–winding cycles can be modulated. As shown in Figure 8, our self-built equipment can be expanded in order to adjust the layer design, wall thickness, and the mechanical performance of the composite pipes.

**Figure 9:**
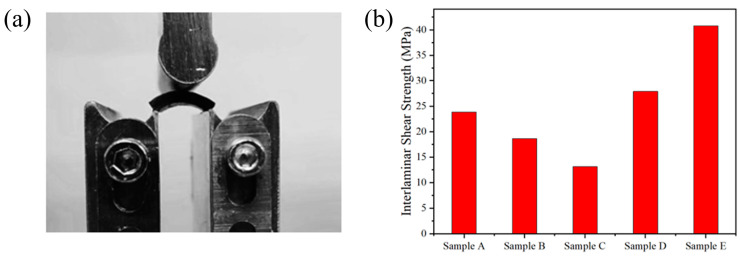
(**a**) Interlayer shear test method. (**b**) Interlayer shear test results.

In order to investigate the effect of pultrusion–winding cycles on the interface bonding performance of composite pipes, different pipes were produced by controlling pultrusion–winding cycles. Interlayer shear tests were conducted on these three types of pipes. The test method is shown in Figure 9a and the test results are shown in Figure 9b. The samples were cut into a width of 10 mm for testing. The center of the sample was placed perpendicular to the indenter, and the experimental speed was 2 mm/min until the sample was damaged. From the analysis of the results, it can be found that the shear strength of Sample A is 23.9 MPa. The shear strengths of Sample B and C are lower than that of Sample A. This is because under the same number of winding cycles, Sample B and C have more axial layers, making it more difficult for heat to conduct, resulting in insufficient resin melting flow and lower interlayer performance. The shear stress–strain curve of Sample D shows that its shear strength is 27.9 MPa. The shear strength of Sample E is 40.8 MPa, which is 70.7% higher than that of Sample A. This proves that the shear strength of pipe fittings also increases with the increase in winding times. This is because multiple winding processes mean more thorough heating and melting of the resin. There is sufficient heat transfer between prepreg tapes, which can make the resin flow more fully, infiltrate the fibers more thoroughly, and cause a longer curing time. Multiple cycles of melt-curing also improve the crystallinity of the resin, thereby better enhancing its mechanical properties.

**Figure 10:**
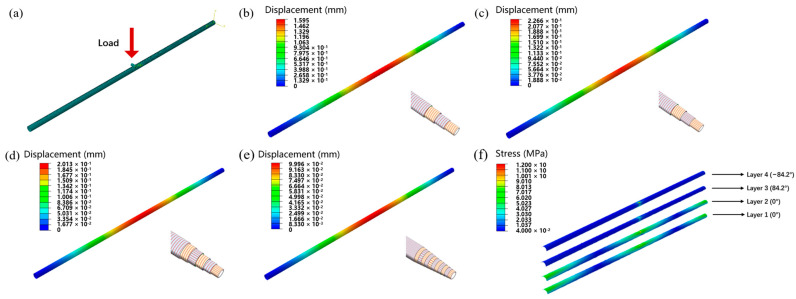
(**a**) Meshing of bending model for pipes. (**b**) Three-point bending-displacement simulation result of circumferential reinforcement pipe. (**c**) Three-point bending-displacement simulation result of Sample A. (**d**) Three-point bending-displacement simulation result of Sample D. (**e**) Three-point bending-displacement simulation result of Sample E. (**f**) Bending stress simulation result of different layers of biaxial reinforced pipe.

In addition, the simulation analysis of three-point bending performance was conducted on conventional winding pipes [84.2/−84.2/84.2/−84.2] and Samples A, D, and E. The bending test method and meshing are shown in Figure 10a. We set boundary conditions, fixed both ends of the pipe fittings completely, and applied a load of 5 N to the middle of the pipe. The simulation results are shown in Figure 10b–e. Based on the simulation results, it can be seen that under the same bending load, Sample A has a smaller displacement compared to conventional winding pipe fittings, and its bending resistance performance is better. With the increase in pultrusion–winding cycles, the displacements of Sample D and Sample E gradually decrease, exhibiting better bending performance. From Figure 10f, it can be seen that the bending stress withstood by the axial layer in Sample A is higher than that of the circumferential layer. The addition of axial pre-impregnated tape effectively improves the bending performance of pipe fittings.

**Figure 11:**
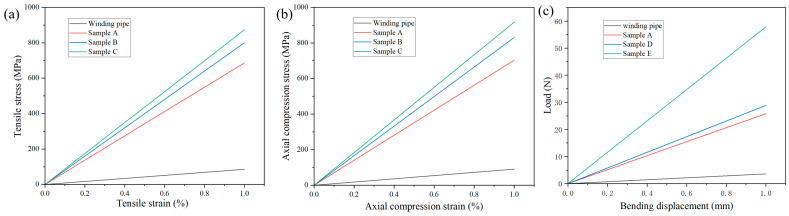
(**a**) Tensile stress–strain simulation curve. (**b**) Axial compression stress–strain simulation curve. (**c**) Bending load–displacement simulation curve.

The mechanical performance can be predicted well based on simulation. Here, the stress–strain curves during compression and tensile testing and the load–displacement curves during three-point bending testing were analyzed through simulation. These curves clearly show the trend of modulus change for different pipes. From Figure 11a,b, it can be seen that the addition of the axial prepreg effectively improves the axial compression and tensile modulus of the pipe fittings. As the number of axial bands increases, the modulus also continuously strengthens. From Figure 11c, it can be seen that the increase in axial prepreg improves the bending resistance of the pipes. With the increase in pultrusion–winding cycles, the bending resistance of the pipe fittings also improves.

### 3.4. Analysis of Compression Failure Forms of Pipes

**Figure 12:**
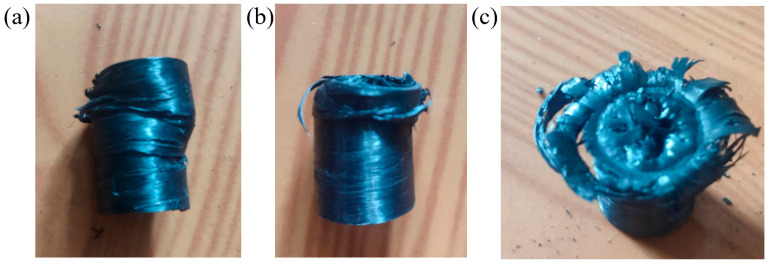
(**a**) Buckling failure of the pipe samples. (**b**) Folding failure of pipe samples. (**c**) Blossom failure of pipe samples.

As shown in Figure 12a–c, there are three main failure modes during the axial compression testing of the pipe fittings. The first form of failure is buckling failure, as shown in Figure 12a. During the axial compression test, the axial prepreg inside the pipe gradually separated from the winding prepreg due to compression, resulting in obvious cracking on the surface of the pipe, deformation on the side of the pipe, and buckling on the surface of the pipe wall. As the experiment progressed, the axial band inside the pipe began to break due to the inability to withstand the load, causing the pipes to twist and deform. This failure mode mainly occurred in the axial compression experiment of Sample C. From the previous test results, it can be seen that the interlayer performance of Sample C is the weakest, resulting in delamination between prepreg and pipe wall buckling.

The second form of failure is folding failure, as shown in Figure 12b. In the initial stage of axial compression test, there may be uneven winding on the wall of the pipe and pores at the connection of the winding tape. The uneven stress on the pipe wall of the pipe leads to stress concentration. With the proceeding of axial compression, the pipe wall begins to exhibit a layered folding form and fails downwards. However, after the pipe wall is damaged, there was no significant axial prepreg detachment or carbon fiber debris. Such forms of damage occurred in Samples A, B, and C.

The third type of failure is Blossom failure, as shown in Figure 12c. In the initial stage of axial compression testing, the internal axial prepreg of the pipe is first subjected to load, compressed, and fractured, bending inward. The prepreg on the pipe wall falls off due to the destruction of the matrix resin, and the layer by layer prepreg is compressed by parallel plates and flipped outward. The composite pipe fittings undergo significant blossom failure. Due to the excellent interface performance between the layers, there was no serious deformation at the lower end of the pipe during the testing process, and there was no instability of the pipe during the testing process. The fiber bundle was compressed and damaged, accompanied by the sound of fiber fracture and matrix damage. This type of damage mode mainly occurred in Samples D and E. Samples D and E have excellent interface performance and have multiple winding forming processes, with a smaller possibility of winding defects.

## 4. Conclusions

In this research, we demonstrate a novel pultrusion-winding technique for thin-walled CF/PEEK composite pipes for on-orbit manufacturing.

- This approach not only achieved bidirectional enhancement of pipes, but also breaks through the size limitation of core mold.
- The parameters including pultrusion temperature, winding speed, winding pressure, and winding angle were optimized, which provided basic experimental data for practical applications.
- The mechanical properties of the pipes including axial compressive strength, circumferential compressive stiffness, and interlayer shear strength were tested for pipes with different axial layers numbers and winding times. It was found that the axial compressive strength could be greatly increased due to the increase in axial prepreg tape layer.
- The interlayer shear strength can be effectively improved due to the increase in pultrusion–winding cycles. The finite element simulation results also proved that the axial fibers share the most axial stress while the circumferential layer greatly decreases the shear strain.

We believe that our self-designed pultrusion-winding approach provides an effective strategy for bidirectional, enhanced, continuous carbon fiber composites pipes with good compressive and shear performance, which offer basic components for the on-orbit manufacturing of large-scale structures in space stations. On the other hand, there is still room for improvement. For example, in our self-built equipment, the mandrel inside the pipe cannot be heated, leading to heavy heat loss in the inner layer. This affects the interface bonding between the inner tapes. Thus, the interface bonding performance between different layers is poor, which limits the mechanical performance of our pipes. And, the cooling speed of the pipes can not be controlled, so it is hard to further study the crystallinity process of the thermoplastic resin during the manufacturing of pipes.

## Figures and Tables

**Table 1:** Basic information on different CF/PEEK pipes.

Type of Pipes	Pipe Layer Design	Wall Thickness (mm)
Sample A	[0_2_/84.2/−84.2]	0.61
Sample B	[0_3_/84.2/−84.2]	0.77
Sample C	[0_4_/84.2/−84.2]	0.92
Sample D	[0/84.2/−84.2]_2_	0.91
Sample E	[0/84.2/−84.2]_4_	1.81

**Table 2:** The simulation input data for materials.

Material Properties	Input Parameters
E1	133,000 MPa
E2	9000 MPa
Nu12	0.28
G12	5100 MPa
G13	5100 MPa
G23	3476 MPa

## Data Availability

Data are contained within the article.
